# Nonadherence to treatment protocol in published randomised controlled trials: a review

**DOI:** 10.1186/1745-6215-13-84

**Published:** 2012-06-18

**Authors:** Susanna Dodd, Ian R White, Paula Williamson

**Affiliations:** 1 Department of Biostatistics, Institute of Translational Medicine, University of Liverpool, Liverpool, L69 3GS, UK; 2 MRC Biostatistics Unit, Institute of Public Health, Robinson Way, Cambridge, CB2 0SR, UK

**Keywords:** Causal effect modelling, nonadherence, non-compliance, trial reporting, trial analysis

## Abstract

This review aimed to ascertain the extent to which nonadherence to treatment protocol is reported and addressed in a cohort of published analyses of randomised controlled trials (RCTs). One hundred publications of RCTs, randomly selected from those published in BMJ, New England Journal of Medicine, the Journal of the American Medical Association and The Lancet during 2008, were reviewed to determine the extent and nature of reported nonadherence to treatment protocol, and whether statistical methods were used to examine the effect of such nonadherence on both benefit and harms analyses. We also assessed the quality of trial reporting of treatment protocol nonadherence and the quality of reporting of the statistical analysis methods used to investigate such nonadherence. Nonadherence to treatment protocol was reported in 98 of the 100 trials, but reporting on such nonadherence was often vague or incomplete. Forty-two publications did not state how many participants started their randomised treatment. Reporting of treatment initiation and completeness was judged to be inadequate in 64% of trials with short-term interventions and 89% of trials with long-term interventions. More than half (51) of the 98 trials with treatment protocol nonadherence implemented some statistical method to address this issue, most commonly based on per protocol analysis (46) but often labelled as intention to treat (ITT) or modified ITT (23 analyses in 22 trials). The composition of analysis sets for their benefit outcomes were not explained in 57% of trials, and 62% of trials that presented harms analyses did not define harms analysis populations. The majority of defined harms analysis populations (18 out of 26 trials, 69%) were based on actual treatment received, while the majority of trials with undefined harms analysis populations (31 out of 43 trials, 72%) appeared to analyse harms using the ITT approach. Adherence to randomised intervention is poorly considered in the reporting and analysis of published RCTs. The majority of trials are subject to various forms of nonadherence to treatment protocol, and though trialists deal with this nonadherence using a variety of statistical methods and analysis populations, they rarely consider the potential for bias introduced. There is a need for increased awareness of more appropriate causal methods to adjust for departures from treatment protocol, as well as guidance on the appropriate analysis population to use for harms outcomes in the presence of such nonadherence.

## Review

### Adherence to prescribed medication

Adherence to long-term therapy has been defined as ‘the extent to which a person’s behaviour - taking medication, following a diet, and/or executing lifestyle changes, corresponds with agreed recommendations from a health care provider’ [1]. Nonadherence to prescribed treatment can take a variety of forms, ranging from not starting treatment at all, to taking treatment at the incorrect time or dose, ‘drug holidays’ (stopping treatment for a period of time before restarting), ‘white-coat compliance’ (improved adherence to treatment regimen around the time of appointment with clinician) and permanent discontinuation of treatment [2]. Nonadherence is a problem affecting many therapeutic areas [3], and has been cited as the primary reason for suboptimal clinical benefit, causing complications of disease, reduced quality of life and wasted health care resources [1]. A vast amount of research has explored its causes [4], and numerous factors related to the patient, disease, treatment, health care provider and health care system are known to be associated with adherence [1]. Despite this, there has been little improvement in adherence rates, with an estimated 1/3 to 1/2 of patients consistently failing to comply in some way with prescribed medications [4], and adherence to lifestyle prescriptions is even lower [3].

### Nonadherence in randomised controlled trials

In a clinical trial setting, treatment adherence implies following the treatment regimen specified in the trial protocol, and is thus dependent not only on the action of the trial participant but also on the cooperation of the treatment provider. Treatment providers may deviate from the randomised treatment protocol when administering or prescribing treatment, by changing the type, dose or schedule of drug prescribed or failing to deliver treatment according to the procedure specified in the protocol (for example, during a surgical operation). Alternatively, the treating clinician may decide over the course of the trial treatment period that it would be in the best interest of the patient to deviate more substantially from the treatment protocol and to instead deliver an entirely different treatment.

Trial participants may also face greater barriers to adherence than those being treated in a usual care setting. Although it is possible that those who agree to take part in a trial may naturally be more motivated to adhere to their prescribed treatment than those in the general population, the trial treatment protocol may be rather more involved or demanding, and may be accompanied by more intense follow-up or invasive assessments than would be experienced in general practice, all of which can hinder participation. Also the uncertainty about the efficacy of a drug being tested in the trial setting may mean that trial participants are less likely to persevere with unpleasant side effects of treatment than those who have been assured of the efficacy of their treatment. Trial participant nonadherence may take the form of premature participant withdrawal from the study (commonly referred to as withdrawal of consent or participant discontinuation of the study) or discontinuation of treatment, either permanently (often referred to as withdrawal from treatment) or temporarily (treatment interruptions). Other forms of nonadherence include failing to follow treatment protocol as regards timing or dosage of randomised treatment, or failing to initiate allocated treatment at all. Thus, compared with a regular clinical setting, an added dimension to the problem of nonadherence arises in clinical trials because of the required adherence of both trial participants and treatment providers to the potentially stringent trial protocol.

### Reporting of adherence information

Clinical trials often fail to report important features of design and analysis that are necessary to ascertain the methodological quality of the trial [5-11]. Lack of adequate trial reporting led to the development (and two further updates) of the Consolidated Standards of Reporting Trials (CONSORT) statement [12-14], which aimed to improve reporting of trial methodology and results by providing authors with a checklist of items recommended for inclusion in trial publications. CONSORT recommends reporting ‘for each group the numbers of participants who were randomly assigned, received intended treatment and were analysed for the primary outcome…Knowing the number of participants who did not receive the intervention as allocated or did not complete treatment permits the reader to assess to what extent the estimated efficacy of therapy might be underestimated in comparison with ideal circumstances.’ Use of CONSORT flow diagrams to illustrate patient flow through the trial has become increasingly common [15], but the information presented in the flow diagram may not be detailed enough to ascertain the true extent and nature of any nonadherence to treatment protocol that may have occurred in a trial.

### Statistical analysis of nonadherence data

When a trial is subject to nonadherence to treatment protocol, the appropriate statistical method of analysis will be depend on the research question of interest. The standard method of analysis by intention to treatment (ITT) classifies participants according to the treatment to which they were originally randomised and ignores information on actual treatment received [16]. ITT is thus useful as an assessment of the trial policy of assigning participants to therapy (also called use-effectiveness or simply the effectiveness of treatment) and should be presented as a primary analysis for all trials, as it reflects the original trial design.

When offered a new treatment, a different question that is likely to be of particular interest to the patient is ‘What treatment effect can I expect if I adhere to treatment?’ This causal effect of treatment may also be of interest to clinicians, health care regulators and pharmacologists [17]. In the presence of treatment protocol nonadherence, however, an ITT does not provide an estimate of the actually administered treatment (also called method-effectiveness or efficacy of treatment [18]) or the causal effect of treatment [19], as the inclusion of nonadherent participants in ITT analysis generally diminishes the estimated treatment effect [16]. Thus, depending on the research question, an ITT approach may need to be supplemented by additional analyses which account for deviations from protocol [17]. In particular, the analysis of harms outcomes using an ITT approach is not recommended because, as in equivalence or non-inferiority trials [16], diminishing the estimate treatment effect in a harms analysis is anti-conservative, making a harmful treatment more likely to be accepted as harmless. As stated by Lewis and Machin [20], ‘a pure ITT approach to the analysis of safety simply adds to the risk of failing to identify potential safety problems, and is therefore never advocated’.

Commonly used methods of analysis to address the issue of nonadherence to treatment protocol and thus provide an estimate of treatment efficacy include per protocol (PP) and as treated (AT) analyses. PP analysis typically excludes or censors participants who deviate from treatment protocol, but exclusion of participants from the analysis in this way can lead to selection bias, as the treatment protocols being compared are likely to present different challenges to adherence and the resultant residual subgroups may not be comparable [21]. Similarly, AT analysis of patients introduces selection bias by simply analysing participants according to treatment received without regard to their randomised allocation.

There exist other statistical techniques to estimate causal effects of treatment and thus account for nonadherence to treatment protocol without introducing biases inherent to PP or AT analyses, such as randomisation-based efficacy estimation methods, which estimate efficacy while taking into account departures from randomised treatment and yet maintaining the balance produced by randomisation [17]. Such analysis techniques are not commonly used, as they tend to be more complex and computationally intensive than the more naive but inherently biased techniques of analyses such as PP and AT, and they necessarily rely on potentially unverifiable assumptions. However, this area of research is growing, and interested readers are directed towards introductory texts such as Bellamy *et al*. [22], Little and Rubin [23] or Greenland *et al*. [24].

### Aims

This review aimed to ascertain the extent and nature of nonadherence to randomised treatment reported in published trials, how such nonadherence is handled in statistical analyses of benefit and harms outcomes, and how well such nonadherence, and the statistical methods to deal with it, are reported in published randomised controlled trials (RCTs).

## Methods

### Terminology

In this article, the term ‘nonadherence to randomised treatment’ is used to indicate any deviation on the part of the patient or treatment provider to the trial treatment protocol, or any treatment change agreed with medical staff but not permitted by the trial protocol. Protocol-permitted treatment changes can also present problems of interpretation but these were not considered in this review. For example, discontinuation of the randomised treatment for safety reasons as specified in the protocol, or premature stopping of a trial according to a predefined stopping rule, were not considered as examples of deviations from treatment protocol in this review.

For the purposes of assessing the adequacy of trial reporting, we have extended the definitions given by Vrijens *et al*. [25] for the three quantifiable phases of patient adherence to prescribed medication (initiation, discontinuation and implementation) for use in a clinical trial setting, where the intervention may be nonpharmacological and where adherence to treatment protocol can be influenced by treatment providers when administering or prescribing treatment as well as by participants, because of the required adherence to the trial treatment protocol.

Thus adherence is defined as the degree of correspondence between a participants’ intended randomised treatment prescription and their actual history of treatment received over the course of the protocol-specified treatment period. Adherence is split into the following phases: initiation of randomised treatment occurs when the participant begins their randomised intervention; discontinuation is defined as the permanent cessation of randomised intervention; the implementation period is the time between these two events. Persistence is defined as perseverance with the randomised treatment as per the trial protocol until the end of the protocol-defined treatment period.

Given that the adequacy of trial reporting on adherence to treatment protocol depends on the duration and complexity of trial-specific interventions, we classified trials according to whether the trial interventions were given at a single or multiple time points. Trials with treatment given at multiple time points (referred to as longitudinal treatment periods) were divided into ‘short-term intervention trials’ involving treatment given at a few discrete time points (for example, single daily dose over the course of a week) or continuously over a short period of time (for example, infusion given for one hour), and ‘long-term intervention trials’.

### Key points to be reported

In order to judge the quality of trial reporting, and the statistical handling of treatment protocol nonadherence, it is important to identify key points that should be explicitly reported in each publication. Table 1 documents the recommendations for explicit reporting of information relating to adherence to treatment protocol, with quoted justification taken from the CONSORT 2010 elaboration document [13] unless stated otherwise. All trials should report the number of randomised participants and the number of participants who initiated their randomised intervention. Longitudinal treatment period trials were also expected to have reported on treatment persistence: in the interest of a conservative assessment of adequacy of reporting, short-term intervention trials were required only to have reported on the number of patients who completed intervention (or the number who discontinued randomised intervention prematurely). Long-term interventions are, however, more likely to result in treatment interruptions or deviations, so patients who were still taking treatment at the end of the protocol-determined treatment period may not have been fully adherent to the treatment schedule for the whole duration of treatment. Thus publications of trials with long-term interventions were judged according to whether they had supplemented their reporting of the number of participants who persisted with the intervention according to the treatment protocol (or conversely the number of participants who prematurely discontinued randomised intervention) with some measure of participant and/or treatment provider adherence to treatment protocol (as appropriate, depending on whether administration of treatment was by the participant or the treatment provider) over the implementation period. Methods used to assess participant or health care provider adherence to the treatment protocol should be described, and clinical justification given for any definition used to define adherence. In particular, clinical trials involving a participant-administered intervention are recommended to use a reliable measure, and ideally a combination of measures, to accurately record participants’ adherence data [26]; thus, in this review, publications of such trials with a long-term intervention period were judged according to whether they collected and reported details of the methods used to assess, and check reliability of, participant adherence. We also recorded whether the use of any adherence threshold to split participants into ‘good’ and ‘poor’ compliers was explained. Although such dichotomisation is not recommended [27], any threshold should be specified in the protocol; otherwise, there may be suspicion that an optimal adherence cut-off has been selected on the basis of the results to allow the most favourable adherence rates to be reported, as the ‘definition of non-compliance is malleable and could be inadvertently manipulated for benefit of investigators’ [28].

**Table 1:** Recommendations for explicit reporting of information relating to adherence to treatment protocol

	**Report for all trials**	**Report according to treatment duration**			**Report reasons for**	**justification**
		**Single (one-off) intervention**	**Short-term intervention**	**Long-term intervention**		
**1. Randomisation**	Randomised^a^					‘Crucial count for defining trial size and assessing whether a trial has been analysed by intention to treat’; necessary to determine whether all trial participants received treatment and were included in analysis.
**2. Adherence to treatment protocol**						
a. Initiation		Initiated (or received) randomised intervention^a^	Initiated randomised intervention^a^	Initiated randomised intervention^a^	Those not initiating randomised intervention	‘Knowing the number of participants who did not receive the intervention as allocated or did not complete treatment permits the reader to assess to what extent the estimated efficacy of therapy might be underestimated in comparison with ideal circumstances.’
b. Completion/persistence^b^			Completed randomised intervention^a^	Persisted with randomised intervention as required by treatment protocol^a^	Those who did not complete/persist with randomised intervention	
**c. Adherence over treatment period**^**c**^						
i) Method			Description of method used to measure adherence over treatment period (and of an additional method to check reliability if trial involves participant-administered intervention)			If participant compliance data are collected, the reliability of the method used to record compliance should ideally be checked by use of another method (for example, treatment diaries backed up by counts of remaining tablets at the end of each course of treatment) [26]
ii) Justification for definition			Justification for any reported definition of adherence (for example, if a threshold is used to define adequate adherence)^d^			‘If patients are to be divided into “compliant” and “noncompliant” groups, the division should ideally be made on the grounds of the relationship of the compliance level to the therapeutic response or outcome’ [29]
iii) Results			Measure of participant and/or treatment provider adherence with randomised intervention (as appropriate)^d^		Any participant or treatment provider nonadherence^d^	
**3. Analysis**						
a. Analysed	Analysed^a^				Any exclusion of participants from analysis	‘Attrition as a result of loss to follow up, which is often unavoidable, needs to be distinguished from investigator-determined exclusion for such reasons as ineligibility, withdrawal from treatment, and poor adherence to the trial protocol…Participants who were excluded after allocation are unlikely to be representative of all participants in the study.’
b. Analysis set composition	How analysis sets differ from randomised groups				Any difference between analysis sets and randomisation groups	‘Erroneous conclusions can be reached if participants are excluded from analysis, and imbalances in such omissions between groups may be especially indicative of bias.’

Quoted justification is taken from CONSORT 2010 elaboration document [13] unless stated otherwise. ^a^Report numbers of participants in each randomised intervention group satisfying condition listed in each cell. ^b^Persistence is defined as perseverance with prescribed treatment until the end of the treatment period. ^c^Adherence is defined as a measure of the degree of correspondence between prescribed treatment and actual treatment received by participant. ^d^Note that, depending on the complexity of treatment, it may not be necessary for trials with a short-term intervention to report on adherence over the treatment period.

Analysis sets for both benefits and harms outcomes should be defined explicitly, with reasons given for the exclusion of any patients from the analysis. If there is any difference between the defined analysis sets and the intervention groups as randomised, this should be stated clearly and any potential resultant bias should be discussed.

### Selection of reports

A search of MEDLINE (using terms randomi$ed controlled trial$ or controlled trial$ or controlled clinical trial$ or RCT$) was carried out in order to identify trial reports published in BMJ, the Journal of the American Medical Association (JAMA), The Lancet and New England Journal of Medicine (NEJM) during 2008. Of the 698 articles obtained from the initial search, 281 (49 from BMJ, 53 from JAMA, 84 from The Lancet and 95 from NEJM) remained after the deletion of duplicates, comments, systematic reviews and meta-analyses. A sample of 100 trial reports were randomly selected from these articles, 16 of which were from BMJ, 20 from JAMA, and 32 each from The Lancet and NEJM.

### Secondary publications

Five of the selected studies were secondary publications of recent trials (original publications were in NEJM [30,31], JAMA [32], American Journal of Medicine [33] and Neurology [34]). In these cases, for the purposes of reporting adherence data, both the primary and secondary publications of the same trial were considered to be the unit of analysis.

### Data extraction

SD recorded the trial publication characteristics relating to the quantitative items listed in Table 1 using a piloted, standardised form. In cases of any doubt or ambiguity, the paper was reviewed by PW.

## Results

### Quality of reporting on departures from treatment protocol

The vast majority of trial reports (96%) included a CONSORT flow diagram*.* Table 2 summarises the quality and completeness of reporting on randomisation, adherence to treatment protocol and analysis in the CONSORT flow diagrams and the text. All 100 trials stated the numbers randomised, but only 58 publications stated how many patients actually initiated their allocated treatment. All trials provided some information on the number of participants included in analysis of the primary outcome, but this information was not always provided for secondary outcomes, particularly when a large number of outcomes was analysed. Forty-three trial reports included an explicit explanation of the composition of the analysis sets used for benefit outcomes, 48 trials labelled the analysis sets (47 ITT and one PP) without further explanation of how the analysis sets were composed, and no details on the composition of benefit outcome analysis sets were given in the remaining nine trials.

**Table 2:** Reporting of key points in 100 trial reports

	**Report for all trials**	**Report according to treatment duration**		
		**Single (one-off) intervention (n = 12)**	**Short-term intervention (n = 22)**	**Long-term intervention (n = 66)**
**1. Randomisation**	100 (100%)	-	-	-
**2. Adherence to treatment protocol**				
a. Initiation	-	10 (83%)	15 (68%)	33 (50%)
b. Completion/persistence	-	-	12 (55%) [17 (77%)]^a^	31 (47%) [51 (77%)]^a^
**c. Adherence over implementation period**				
i) Method	-	-	1 (50%)^b^	21 (47%)^b^
ii) Justification for definition	-	-	0 (0%)^c^	0 (0%)^c^
iii) Results	-	-	5 (23%)^d^	22 (33%) [28 (42%)]^d^
**3. Analysis**				
a. Number analysed	100 (100%)^e^	-	-	-
b. Analysis set composition	43 (43%) [91 (91%)]^f^	-	-	-

Cells left blank are not required. ^a^Number (%) of trials that fully reported [partially reported] on persistence or completion of randomised treatment. ^b^Number of short-term (n = 2) or long-term (n = 45) intervention trials with patient-administered treatment (2) is used as denominator for %. ^c^Number of short-term (n = 1) or long-term (n = 18) intervention trials with patient-administered treatment that reported adherence definition is used as denominator for %. ^d^Number (%) of trials that fully reported [partially reported] some measure of nonadherence on the part of patient or treatment provider. ^e^Number (%) of trials that reported the number included in analysis of primary outcome. ^f^Number (%) of trials that fully reported [partially reported] analysis set composition; partially reporting trials stated that analysis was by intention to treat (n = 47) or per protocol (n = 1) but did not explicitly explain composition of analysis sets.

Table 3 provides a breakdown of persistence and adherence information reported in the 88 studies with longitudinal treatment periods. The majority (81, 92%) provided some information on treatment completeness, but this was sometimes incomplete or vague.

**Table 3:** Breakdown of persistence and adherence reporting in 88 trials with longitudinal intervention periods

		**Short-term intervention (n = 22)**	**Long-term intervention (n = 66)**	**Total**
**Persistence**				
Fully reported^a^		12	31	43
Partially reported only^b^		5	20	25
Partially reported, including reporting the number of participants who:				
	Withdrew	1	3	4
	Withdrew consent	1	2	3
	Lost to follow-up (during treatment)	-	3	3
	Lost to follow-up (unclear whether during treatment)	1	1	2
	Discontinued due to certain event(s)^c^	-	6	6
	Completed study	3	8	11
	Discontinued study	-	2	2
	Completed different aspects of treatment protocol (reported separately)^d^	-	2	2
	Completed treatment in trial overall (not by treatment group)	1	1	2
Not reported		5	15	20
**Adherence over implementation period**				
Fully or partially reported, including reporting^b^		5	22 (6)	27 (6)
	Average measure of participant adherence^e^	2	20 (6)	22 (6)
	Average measure of adherence on part of treatment provider	3	1	4
	Treatment interruptions	-	2	2
Not reported		17	38	55
**Overall reporting**				
Some reporting		19	62	81
	Persistence reported only (fully or partially)	14	34	48
	Adherence reported only (fully or partially)	2	11	13
	Both persistence and adherence reported (fully or partially)	3	17	20
Not reported		3	4	7

^a^Reported the number of participants still taking treatment at end of treatment period, or the number who completed treatment, or who discontinued or withdrew from randomised intervention prematurely. ^b^Trial publications may have reported on one more than one of the categories listed. ^c^Reported numbers discontinuing only for one reason (for example, adverse events). ^d^Unable to discern how many participants received entire intervention. ^e^Figure in brackets indicates number of publications reporting adherence measure for overall trial or combinations of intervention groups, not by individual intervention group.

Overall, reporting of treatment initiation and completeness was judged to be adequate in only 7 (11%) of 66 trials with long-term interventions (requiring reporting of treatment initiation, persistence and some measure of adherence over the implementation period) and 8 (36%) of 22 trials with short-term interventions (requiring reporting of treatment initiation and completion). Reporting of treatment receipt was judged to be sufficient in 10 (83%) of 12 trials with a one-off treatment.

Thirty-three trials with the intervention given at multiple time points reported information on treatment interruptions (two trials) or a measure of average adherence over the treatment period, on the part of the participant (28 trials) and/or treatment provider (four trials; one trial reported on adherence of both the participant and treatment provider), but six of these reported this information for the whole trial or combinations of intervention groups rather than by individual intervention group. Reported measures included the percentage of patients who achieved a particular arbitrary level of adherence in terms of the proportion of doses received or the proportion of time patients were supplied with randomised drug over the course of the trial.

Less than half (21, 47%) of the 45 trials with participant-administered treatment mentioned checking adherence, and only five of these assessed the reliability of the adherence data using a second method. The most common methods to ascertain participant adherence involved counting pills (11 trials) or questioning the participant in some manner (10 trials), even though these methods are not considered to be particularly reliable [26]. More accurate methods, such as medication events monitoring systems or measurement of drug metabolite or marker in bodily fluids, were not used, except in one trial that used blood tests. Nineteen trials (17 of which were drug trials) explicitly defined adherence in terms of the proportion of intervention received (or proportion of time supplied with drug) or discontinuation of intervention. Thirteen of these specified a threshold to define adequate adherence (ranging from 50% to 100% but most commonly 80% (four trials) or two-thirds (three trials)) but no report included an explanation for the choice of thresholds.

Twelve (34%) of the 35 trials of nonpharmacological interventions mentioned that adherence of treatment providers was monitored, four of which reported quantitative results.

### Ambiguities in trial reports

Commonly used terms in CONSORT flow diagrams alluding to nonadherence (or adherence) to treatment protocol such as ‘discontinued’, ‘completed study protocol’, ‘withdrew’, ‘protocol deviations’ and ‘loss to follow-up’ do not provide explicit information on completeness of treatment unless accompanied by clarification on timing or treatment actually received. For example, the term withdrew can indicate withdrawal from treatment only, withdrawal from further follow-up or withdrawal of consent regarding inclusion of a patient’s data in the study. In 13 (62%) of the 21 trials that included the term withdrew in the flow diagram, it was not possible to ascertain whether the participants who withdrew had actually initiated treatment before withdrawing. Similarly, the timing of withdrawal was not clear in 8 (38%) of the 21 trials that described participants who ‘withdrew consent’ in the flow diagram.

Fifteen (18%) of the 85 trials with a longitudinal treatment which presented a flow diagram referred to the number of participants who ‘received’ when they meant ‘initiated’ treatment; this could potentially mislead readers as use of the word received may be incorrectly interpreted as receipt of the entire intervention.

Three trials with a long-term intervention period referred in the CONSORT diagram (and four more referred in the text) to the number of patients who ‘completed’ treatment when it would have been more accurate to report this figure as the number ‘still on treatment at end of treatment period/trial follow-up’, as completion of treatment may imply complete adherence to, as well as persistence with, randomised intervention throughout the treatment period.

### Extent and nature of nonadherence to treatment protocol reported

Ninety-eight publications reported at least one form of departure from treatment protocol (see Table 4)*.* Direct comparison of the extent of departure from treatment protocol across trials is not straightforward, as trials differed greatly in terms of type and duration of intervention, definitions used to define nonadherence and level of reporting. However the distribution of percentage of patients displaying some form of deviation from treatment protocol (or the average degree of nonadherence in a single trial), based on information reported in the trial publications, can also be seen in Table 4.

**Table 4:** Reported forms of departure from treatment protocol

	**Number (%)**
**Reported some form of nonadherence to treatment protocol**^**a**^	98
Participants who did not initiate allocated treatment	39
Participants with incomplete treatment (among those who initiated allocated treatment)	78
Participants who switched trial treatments	12
Participants who started open label treatment (not as per protocol)	7
Participants who started disallowed or non-trial treatment	4
Evidence of contamination between treatment groups	3
Other forms of nonadherence to treatment dose or schedule	23
Nonadherence on the part of treatment providers	12
**Did not report any nonadherence to treatment protocol**	2
**Percentage of patients experiencing or displaying some form of nonadherence to treatment protocol**	
None reported	2
0 to 5%	23
5 to 10%	10
10 to 20%	22
20 to 30%	11
30 to 50%	12
>50%	9
>0% but unclear^b^	11

^a^Trial publications may have reported the number of participants in more than one of the categories listed. ^b^For example, trial report states only that the treatment providers were not adherent to some degree, or it was not possible to distinguish withdrawal from treatment for legitimate reasons (for example, death or treatment changes permitted by protocol following adverse events) from withdrawal due to nonadherence.

### Handling of departure from treatment protocol in statistical analyses

#### *Benefit outcomes*

Of the 98 trials that reported some form of nonadherence to treatment protocol, 51 (52%) reported some form of analysis method to handle such nonadherence in the analysis of benefit outcomes (see Table 5). Forty-six trials adjusted for treatment protocol deviations by carrying out analysis based on PP analysis (total not shown in Table 4), by censoring or excluding participants who had violated the treatment protocol in some way, but half of these analyses were labelled as ITT or modified ITT analyses. Only one trial [35] aimed to address the bias introduced from potentially informative censoring of patients at the point of deviation from treatment protocol, by weighting their censoring by the inverse of their estimated probability of adhering, as advocated by Robins and Finkelstein [36]. Other analysis methods that dealt with departures from randomised treatment included treating discontinuation of treatment (or starting disallowed or rescue medication) as a treatment failure in analysis (three trials), AT analyses (analysing participants according to the actual treatment received regardless of randomisation allocation) (three trials) and analysing outcomes concerned with time to discontinuation of trial drug (four trials).

**Table 5:** Statistical methods addressing nonadherence to treatment protocol

	**Number (%**^**b**^**)**	**Definition of analysis set**	**Number**
**Reported a statistical method addressing nonadherence to treatment protocol**^**a**^	51 (52)		
Variant of PP			
	Primary PP analysis described as ‘PP’		18
		Included only those participants who received full randomised intervention	8
			2
		Minimum degree of adherence required	5^c^
		Included only those taking treatment at particular time during trial	1
		Excluded participants if they started disallowed medication	1
			1
	Primary PP analysis described as ‘ITT’ or ‘modified ITT’ analysis		23
		Included only those participants who received full randomised intervention	1
		Included only those participants who received at least one dose of randomised intervention	16
		Included only those participants who received the single treatment	3
		Excluded participants if they deviated from treatment protocol	3
	Sensitivity analysis		12
		Included only those participants who received full randomised intervention	4
		Excluded participants if they received disallowed treatments	2
		Minimum degree of adherence required	2^d^
		Censored participants at the point of deviation from treatment protocol	4^e^
	Inverse probability of censoring weighted method		1^f^
	Subgroup analysis		2^g^
	Unlabelled analysis		1^h^
As treated analysis			3
Discontinuation of treatment analysed as treatment failure			3
Time to treatment discontinuation included as trial outcome			4
**Did not report a statistical method to address nonadherence to treatment protocol**	47 (48)		

^a^Nine trials carried out two methods of analysis, two trials carried out three methods and one trial carried out four methods. ^b^Number of trials reporting some form of nonadherence (98) is used as denominator for %. ^c^Adherence thresholds used were 60%, 2/3, 75%, 80% and 90%. ^d^Adherence thresholds used were 2/3 and 5/6. ^e^Censoring times: time of starting disallowed intervention, when participants reported taking less than 2/3 of their medication in the past year, when received treatment out of trial, or censored following six-month lag after receiving less than 80% of drug. ^f^Censored when received <80% of drug, weighted by the inverse probability of each participant’s estimated adherence probability. ^g^Analysis split into two groups (according to whether participants had taken more or less than 50% of the prescribed medication) in one trial and into three groups (according to the proportion (>90%, 75-90% or <75%) of their time at risk that they were supplied with drug) in other trial. ^h^Included if received at least one dose of treatment. ITT: intention to treat; PP: per protocol.

Of the 20 trials where adjustment for nonadherence was explicitly compared with other analyses, four trials reported that adjustment for nonadherence resulted in more extreme treatment effects; in the remaining 16 trials it was not reported to have made a substantial difference to conclusions.

#### *Harms outcomes*

Of the 69 trials that presented a harms analysis, 43 (62%) did not define the specific population set that was used in this analysis (see Table 6). Of the 26 trials that specifically defined a harms analysis population, the majority (18, 69%) specified that analysis was based on actual treatment received and included all patients who had received at least one administration of study agent, but only one study specifically stated that participants who received the alternative treatment rather than that allocated to them would be included in the alternative treatment group for this analysis. The remaining 43 trials that did not define a specific harms analysis population most commonly appeared to analyse the harms outcomes according to the specified benefit analysis population (31 ITT, 2 PP).

**Table 6:** Harms analysis populations in 69 trials that presented harms analyses

	**Number (%**^a^**)**	
**Harms analysis population specifically defined in methods**	26 (38)	
**Defined harms analysis population**	Based on actual treatment received (that is, including all patients who had received at least one administration of treatment)	18
	Intention to treat	5
	All who started allocated treatment	2
	All who completed allocated treatment	1
**Harms analysis population not specifically defined in methods**	43 (62)	
**Inferred harms analysis population**	Stated ‘safety population’ without further definition	1
	Apparently analysed as per efficacy outcomes	33
	Intention to treat definition	31
	Per protocol definition	2
	No details given of harms or benefit analysis population	9

^a^The number of trials with harms analyses presented (69) is used as the denominator for %.

## Discussion

### Reporting of adherence information

We found that the vast majority of RCTs are subject to at least one form of nonadherence to treatment protocol, most commonly incomplete treatment or non-receipt of allocated treatment, but even the most basic adherence information on initiation, completion and premature discontinuation of treatment was not presented in some trials. Perhaps most remarkable was the fact that 42% of the publications did not explicitly state how many patients actually initiated their randomised treatment. The template for the CONSORT flow diagram suggests that, in the treatment allocation box, trialists should report the number of participants who ‘received’ the allocated intervention, and in the follow-up box, they should then report the number who ‘discontinued’ the intervention. However, except in the case of trials with treatment given at a single time point (only 12% of the trials in this review), it would be more accurate and less misleading to ask trialists to report the number of participants who ‘initiated’ rather than ‘received’ the intervention, as ‘initiated’ is unambiguous but ‘received’ may be interpreted either as initiation or as receipt of the entire study treatment. Indeed, in the 2010 CONSORT elaboration document, the table which details the information required in the flow diagram (Table 3) states that the treatment allocation box should include the number of participants who ‘completed’ treatment as allocated, rather than ‘initiated’ or at least ‘received’.

The absence of any explicit reporting of the number of participants who initiated intervention may lead readers to assume that all randomised participants at least started their randomised intervention. Indeed Vrijens *et al*. [25] state that, in the context of clinical trials of prescribed medication, given that the first dose of a randomised medication is usually administered on site following informed consent, ‘it is often assumed that initiation is implicit for all included patients’. However, more than one-third (26, 38%) of the 69 drug trials in our review included participants who did not initiate their randomised intervention.

Other inadequacies in reporting related to departures from the treatment protocol were evident in this review. For example, none of the 13 trial reports that specified a cut-off to define adequate adherence included an explanation for the choice of threshold. As discussed by Vrijens and Urquhart [27], the use of *ad hoc* threshold values, such as the ‘often-used but never pharmacometrically justified’ adherence criterion of taking at least 80% of prescribed doses to define the sufficient exposure to drugs needed to achieve satisfactory therapeutic results, is unacceptable, as such a threshold will depend on a wide range of underlying drug-, disease- and formulation-specific pharmacodynamics.

### Statistical methods to deal with departures from treatment protocol

Although more than half (51 out of 98, 52%) of the trials that were subject to nonadherence to treatment protocol implemented a statistical analysis method to deal with such nonadherence when analysing benefit outcomes, these were most commonly based on variations of PP analysis (46 trials) and very few recognised, or sought to address, the potential for bias introduced when excluding or censoring patients at the point of deviation from the treatment protocol. One trial [35] sought to address the potential bias caused by censoring of patients at the point of deviation from the treatment protocol, using the inverse probability of censoring weighted method [36], and one other trial [37] provided justification for the decision to exclude participants who had not received the allocated intervention, citing Fergusson *et al*. [38] and stating that the ‘omission [of such patients] would be equally distributed between groups, would be unrelated to treatment assignment, and would not bias outcome ascertainment’. Trial publications commonly presented insufficient detail or explanation on the analysis sets used for benefit and harms outcomes. The composition of analysis sets for their benefit outcomes were not explained in 57% of trials, and 62% of trials that presented harms analyses did not specifically define harms analysis populations. This common lack of discussion regarding whether, and if so why, patients were excluded from analysis and the potential biases which may result from such exclusions indicates a low priority given to the issue of nonadherence to treatment protocol in the analysis of published trials.

### ITT analysis to handle nonadherence to the treatment protocol

Seventeen trials carried out what were referred to as ITT analyses but the ITT analysis set excluded participants if they did not adhere to the treatment protocol in some manner. This mislabelling of ITT analyses has been noted in previous reviews of ITT analyses in recent trial publications [39-42]. Comparison of previous reported results with those here suggests that the use of the phrase ITT to describe analyses of RCTs is becoming more common (48% in 1999 [41], 71% in 2007 [39] and 83% in 2008), at least in these four leading medical journals, but that the correct definition of ITT analysis is being followed less commonly: 13% of ITT trials published in 1999 excluded participants who did not receive the randomised treatment [41] compared with 22% of such trials in our review (*P* = 0.086, Pearson’s χ_1_^2^ statistic = 2.95).

One reason for the exclusion of participants who did not receive treatment in ITT analyses may be the fact that more relaxed definitions of ITT have been advocated in the past. For example, Gillings and Koch [43] considered that, under most circumstances, the ITT population can be defined as all randomised patients who are known to have received at least one dose of treatment and who provide follow-up data for one or more of the key efficacy variables. Also the International Conference on Harmonisation of Technical Requirements for Registration of Pharmaceuticals for Human Use Good Clinical Practice (ICH GCP) E9 guidelines [16] state that ‘it may be reasonable to eliminate from the set of all randomised subjects those who took no trial medication. The ITT principle would be preserved despite the exclusion of these patients provided, for example, that the decision of whether or not to begin treatment could not be influenced by knowledge of the assigned treatment.’ (Note that only one trial in this review provided any justification for the exclusion of such subjects.)

The 2001 CONSORT statement may inadvertently have contributed to the incorrect use of the term "ITT analysis" that was observed in our cohort of 2008 publications, and as such the phrase asking ‘whether the analysis was by “intention to treat”’ in the 2001 version of CONSORT has been amended to ‘whether the analysis was by original assigned groups’ in the 2010 version.

### Harms analyses

A variety of analysis sets were used for the analysis of harms data in this cohort of trials, and this may be a consequence of the lack of consensus in the research literature on the appropriate harms analysis population to be used in the event of departures from treatment protocol. The 2001 version of the CONSORT statement [12] supports the view of Lewis and Machin [20] that ITT is not appropriate for analysis of harms outcomes. Similarly, the ICH GCP guidelines [16] suggest that analysis of harms data should be according to treatment received, that is, all participants who received at least one dose of a treatment should be included in that treatment group for harms analyses. This was the most common analysis set specifically defined for harms outcomes in this review.

However, the 2004 CONSORT extension for reporting harms [44] conversely states that ITT is usually preferred for both benefit and harms outcomes because it reflects the original trial design. The CONSORT 2010 statement has removed any reference to the appropriateness or otherwise of ITT analysis populations for harms outcomes.

The variation in the analysis populations chosen for harms outcomes in the presence of treatment protocol nonadherence suggests that explicit guidance is needed on how harms data from patients who deviate from treatment protocol should be analysed*,* for example, what to do if a patient receives both or none of the trial treatments, or if they receive a treatment to which they were not randomised.

### Importance of collecting adherence data in RCTs

Although ITT analysis is an important part of any trial, there is a potential danger that the spirit of ITT could be interpreted as an indication that collecting or reporting data on the degree of adherence to the treatment protocol is unimportant. However, even when there is no intention to formally investigate the relationship between treatment uptake and outcome, reporting information on the degree of intervention received is arguably important to assess the degree to which the intervention is even reaching the targeted population. Otherwise it may not possible to judge whether an unfavourable observed effect of treatment, for example, may be in part due to non-receipt rather than ineffectiveness of the treatment, and if it is due to non-receipt of the intervention, whether steps could be taken to improve uptake of the treatment to potentially enhance treatment effect. Indeed, an ITT analysis can only provide information on the effectiveness of the intervention as it was implemented in the trial (for example, the trial policies on treatment changes) and according to the level of adherence observed in the trial. In reality, the implementation of the intervention in clinical practice may differ, and adherence rates may improve (or diminish) directly because of dissemination of results from the trial itself and resultant changes in expectation of the intervention amongst users and clinicians. Thus, even the interpretation of the effectiveness of an intervention may be limited from an ITT analysis. Collecting information on adherence and treatment changes that occurred in the trial could potentially allow subsequent statistical investigation into the impact of changes to administration of the treatment or adherence rates on the treatment effect [45]. Even in the case of so-called large, simple trials, where emphasis lies on the collection of limited data from a very large number of participants [46], collection of adherence information from just a small random subsample of patients may add to the clinical interpretation of trial results without greatly increasing the burden on participants or trial staff.

Another argument for reporting adherence information in trial publications is the need to consider variation in adherence rates across trials in a systematic review. Such variation is a potential source of heterogeneity even when true treatment effects are identical across trials [19], as the choice of statistical analysis method in the presence of departure from randomised treatment can affect trial results and conclusions. The issue of whether these differences in adherence rates should be considered as a source of bias by statistical reviewers depends on the purpose of the systematic review. If, as is commonly the case, a systematic review aims to estimate the effectiveness of a treatment in order to inform decisions about health care policies, the issue of variable adherence rates may not be considered a biasing factor. Conversely, if the systematic review is focussing on an estimation of treatment efficacy, it is unlikely that reviewers will be able to appropriately allow for nonadherence without the availability of individual patient data. However, given that choices made in the statistical handling of nonadherence in trial publications can affect trial outcomes and conclusions, it is important whatever the research question for systematic reviewers to consider whether differing degrees of adherence in trials may be causing heterogeneity.

### Increased interest in causal analysis

Ten trial reports in this review specifically discussed the difficulty in interpreting trial results in the presence of treatment protocol nonadherence (although only seven of these trials actually carried out analyses that in some way investigated the influence of the nonadherence to treatment protocol). In addition, two cancer trials discussed the related problem of how to interpret ITT analysis in the face of substantial treatment changes that, rather than being caused by nonadherence to treatment protocol, are actually permitted by the protocol. In the case of late stage cancer trials, it is common for treatment protocols to allow patients to switch treatments or receive additional salvage therapy at the point of disease progression or relapse. This is a particular ethical requirement in placebo-controlled trials, but is also becoming more common in trials comparing active treatments, as a result of the increasing number of second- and third-line therapies that are becoming available [47]. Although such trial designs do not impact on the interpretation of the commonly used outcome progression-free survival (defined as time to progression or death), the analysis of overall survival is complicated by the resultant merging of the treatment experience of the randomised groups following progression, leading to a diminished effect. Consideration of overall survival is particularly important for patients and policy makers, and thus interest is growing in how to address the issue of treatment switches in order to provide unbiased and clear treatment comparisons which are not available from ITT, PP or AT analyses. Causal effect modelling techniques are receiving attention as a potential solution to the problem, and have recently been implemented to guide decision makers [48-50].

### Limitations

This review is limited by a sample size of 100 trial publications taken from only four high impact general medical journals, and thus the generalisation of our results to other less widely read or more subject-specific journals may be limited. However we would argue that, for this reason, the findings evident from this review should be regarded as an estimate of the upper limit of the quality of reporting and analysis of nonadherence to treatment protocol. As regards the critique of analysis methods, because we did not contact trial authors directly, we cannot be sure that the details reported in the trial publications were complete and accurate accounts of how analysis proceeded. As we did not have access to the protocols or statistical analysis plans for the trials in this review, it was not possible to ascertain whether the analyses carried out to examine the effect of treatment protocol deviations were decided prior to data collection or were *post hoc* decisions. Two trials admitted that the statistical method to deal with departures from treatment protocol were *post hoc* analyses.

The main limitation of this review was the use of a single reviewer for data extraction, although this reviewer was able to consult the opinion of a second reviewer whenever there was doubt as to appropriate classifications. This was necessary, however, in less than 5% of the articles reviewed, as systematic data extraction from all trial reports was undertaken using clear definitions for classification of results.

## Conclusions

Nonadherence to treatment protocol is widespread among trials and is recognised by some trialists as potentially obscuring treatment efficacy, but statistical methods of analysis to handle such nonadherence typically exclude or censor participants who deviate from the treatment protocol, without discussion of the potential bias introduced in such an analysis. Nonadherence information presented in trial reports can be ambiguous or scant, particularly relating to treatment initiation and completeness. Reporting information on the uptake and acceptance of treatment is important for the interpretation of the success of the trial treatments, for clinicians, patients and health policy decision makers, even when the analysis does not aim to adjust for nonadherence to treatment protocol. Trialists need to be more aware of the importance of reporting the degree of adherence to the treatment protocol.

### Recommendations box for trialists

1. Trialists should document in the trial protocol how they plan to measure and report nonadherence to treatment protocol, with an explanation in protocol and publication of why the chosen adherence measure is clinically the most important and relevant.

2. The decision on whether, and how, to examine the effect of nonadherence to treatment protocol should be made prior to data collection, and details of the planned analyses should be documented in the protocol (and statistical analysis plan) with an explanation for proposed methods. Trial publications should include a discussion of potential bias introduced by any such analyses, and efforts should be made in the statistical analysis to reduce any bias introduced by excluding participants from analysis.

3. Composition of analysis sets used for benefit and harms outcomes should be defined explicitly, rather than merely labelled as ITT or PP.

4. The total numbers of participants who were randomised, excluded (with reasons) and analysed for each outcome should be reported as recommended in the CONSORT statement [13].

5. Potentially ambiguous phrases such as ‘protocol deviations’, ‘completed study/treatment protocol’, ‘withdrawal’, ‘intention to treat’, ‘modified intention to treat’, and ‘per protocol’ should be carefully defined in terms of the treatment protocol if used in trial reports.

6. Trial reports should clearly distinguish between withdrawal from treatment and withdrawal from study: that is, they should clarify whether participants withdrew from treatment but agreed to continuing to provide follow-up data or whether participants also withdrew from further follow-up, and if the latter, whether or not they consented for the data collected up to the point of withdrawal to be included in the analysis.

7. Reporting of treatment receipt and completeness should reflect the duration of trial intervention(s):

a. Trials with the intervention given at a single time point should report the number of participants who received the allocated intervention in each randomised group.

b. Trials with a short-term intervention should report the number of participants initiating and completing allocated treatment as specified in the protocol in each randomised group.

c. Trials with a long-term intervention should report the number of participants initiating and persisting with allocated treatment as specified in the protocol, along with a measure of participant and/or treatment provider adherence (as appropriate) over the treatment period, in each randomised group.

8. Participant adherence should usually be assessed in at least a random subsample of individuals in trials involving participant-administered treatment using a reliable method or, if necessary, using more than one method to gauge the reliability of the assessment.

9. A biological or medical explanation should be provided for any thresholds used to define adequate adherence, and these should be specified in the trial protocol.

## Abbreviations

AT: as treated; CONSORT: Consolidated Standards of Reporting Trials; ICH GCP: International Conference on Harmonisation of Technical Requirements for Registration of Pharmaceuticals for Human Use Good Clinical Practice; ITT: intention to treat; JAMA: Journal of the American Medical Association; NEJM: New England Journal of Medicine; PP: per protocol.

## Competing interests

The authors declare that they have no competing interests.

## Authors’ contributions

SD developed the protocol, carried out the search and data extraction, and drafted the manuscript. PW conceived the initial idea, helped to develop the protocol, acted as a second opinion on data extracted and commented on drafts of the manuscript. IW helped to develop the protocol and commented on drafts of the manuscript. All authors read and approved the final manuscript.
